# B cell depletion therapies in autoimmune diseases: Monoclonal antibodies or chimeric antigen receptor-based therapy?

**DOI:** 10.3389/fimmu.2023.1126421

**Published:** 2023-02-10

**Authors:** Zheng Zhang, Qian Xu, Liang Huang

**Affiliations:** ^1^ Department of Orthopedics, Tongji Hospital, Tongji Medical College, Huazhong University of Science and Technology, Wuhan, China; ^2^ Department of Hematology, Tongji Hospital, Tongji Medical College, Huazhong University of Science and Technology, Immunotherapy Research Center for Hematologic Diseases of Hubei Province, Wuhan, China

**Keywords:** autoimmune disease, B cell depletion, monoclonal antibody, chimeric antigen receptor, immunotherapy

## Abstract

Immune system detects foreign pathogens, distinguishes them from self-antigens and responds to defend human body. When this self-tolerance is disrupted, the overactive immune system attacks healthy tissues or organs and the autoimmune diseases develop. B cells and plasma cells contribute a lot to pathogenesis and persistence of autoimmune diseases in both autoantibody-dependent and autoantibody-independent ways. Accumulating data indicates that treatments aiming to eliminate antibody-secreting cells (B cells or plasma cells) are effective in a wide spectrum of autoimmune diseases. Monoclonal antibodies (mAbs) deplete B cell lineage or plasma cells by signaling disruption, complement-dependent cytotoxicity (CDC) and antibody-dependent cellular cytotoxicity (ADCC). Engineered-T cells armed with chimeric antigen receptors (CARs) have been adopted from field of hematological malignancies as a method to eliminate B cells or plasma cells. In this review, we update our understanding of B cell depletion therapies in autoimmune diseases, review the mechanism, efficacy, safety and application of monoclonal antibodies and CAR-based immunotherapies, and discuss the strengths and weaknesses of these treatment options for patients.

## 1 Introduction

The immune system defends human body by a tightly controlled network that detects foreign pathogens, distinguishes them from self-antigens and responds (1). When this self-tolerance is disrupted, the immune system accidentally attacks our bodies instead of protecting them that results in development of autoimmune diseases (2). There are over 100 known autoimmune diseases, among which lupus, rheumatoid arthritis (RA) and multiple sclerosis (MS) are common ones. Multiple populations of immune cells are involved in the pathogenesis of autoimmune diseases. Adaptive immune cells, especially B cells and T cells are confirmed to be primary contributors to the overactive immune response (3). In recent decades, evidence accumulated that B cells contribute to pathogenesis and persistence of autoimmune diseases in both autoantibody-dependent and autoantibody-independent ways.

Activated B cells differentiate into antibody secreting cells (ASCs) in lymph nodes and spleen, and then transiently circulate in the blood or migrate to the bone marrow. The autoantibodies secreted by ASCs contribute to autoimmune diseases through modulating important pathways, initiating immune-complex-mediated inflammation and depleting specific types of cells (4–8). Besides antibody secretion, B cells involved in multiple biologic processes, including antigen presentation, cytokine production, regulatory B cells (Bregs) dysfunction, T cell activation and polarization and organization of other inflammatory cells. They can internalize immune complexes and present selected peptides to CD4^+^ T cells in the context of major histocompatibility complexes II (MHCII) (9). Bregs were found functionally deficient in patients with SLE which had a defective CD40 response and impaired IL-10 production (10). Moreover, B cells can produce numbers of cytokines influencing autoimmune pathology including IL-6, TNF, IL-10, IFN-γ, etc. (11) and contributing to the cytokine environment leading to primary T cell polarization. This T cell and B cell cognate interaction is important for the pathogenesis of autoimmune diseases.

Therefore, depletion of B cells has been considered as treatment for autoimmune diseases since 1990’s and many therapies aiming to eliminate B cells were exploited and applied. Monoclonal antibodies (mAbs) and chimeric antigen receptor T (CART) cell therapies have shown encouraging results in a wide range of B cell malignancies. Rituximab was the first mAb approved in 1997 by U.S. Food and Drug Administration (FDA) for the treatment of relapsed or refractory CD20-positive, B-cell, low-grade or follicular non-Hodgkin’s lymphoma (NHL) (12). For its satisfying efficacy and safety, rituximab containing immunochemotherapies are widely used in B-cell NHL and becoming the standard of care. CART cells, one of the most successful immunotherapies have been approved by FDA in 2017 as a treatment of refractory pre-B cell acute lymphoblastic leukemia and diffuse large B cell lymphoma (13). The high efficacy of CART cell therapy solidified adoptive cell therapies as the “third pillar” of medicine along with small-molecule drugs and biologics (14). Many research teams aim to extend the applications of these two types of immunotherapies to as many autoimmune disease types as possible. This paper will review the mechanism, efficacy, safety, and application of mAbs and CART immunotherapy in use and discuss the strengths and weaknesses of these treatment options for patients.

## 2 Mechanism of monoclonal antibody therapy in autoimmune diseases

Antibody binds to the antigen, ligand or receptor that are expressed on the surface of B cells, and disrupts the downstream signaling pathways associated with cellular growth, proliferation or apoptotic mechanisms (15). Depending on different antigens, the corresponding mAbs induce the apoptosis of target cells by different mechanisms. For example, CD20 is a part of a cell-surface complex responsible for adjusting calcium transport. When antibodies bind to CD20, changes in Ca^2+^ concentration are induced and capable of controlling cell growth and apoptosis in B cells (16). In addition to signaling inhibition to induce apoptosis of target cells directly, mAbs also can eliminate them indirectly *via* antibody-dependent cell-mediated cytotoxicity (ADCC), by which the mAbs recruit immune effector cells with cytotoxic properties like natural killer (NK) cells, monocytes, macrophages, and polymorphonuclear leukocytes to kill the antigen-expressing cells (17, 18). Some mAbs such as rituximab and ofatumumab, induce complement-dependent cytotoxicity (CDC) as well (19, 20). Cascade of complement proteins are activated and form a complex to attack the membrane of target cells when C1 complex binds the antigen-antibody complex. mAbs applied in autoimmune diseases usually utilize more than one mechanism in their pharmacological actions.

## 3 Targets of monoclonal antibodies in autoimmune diseases

**Figure 1:**
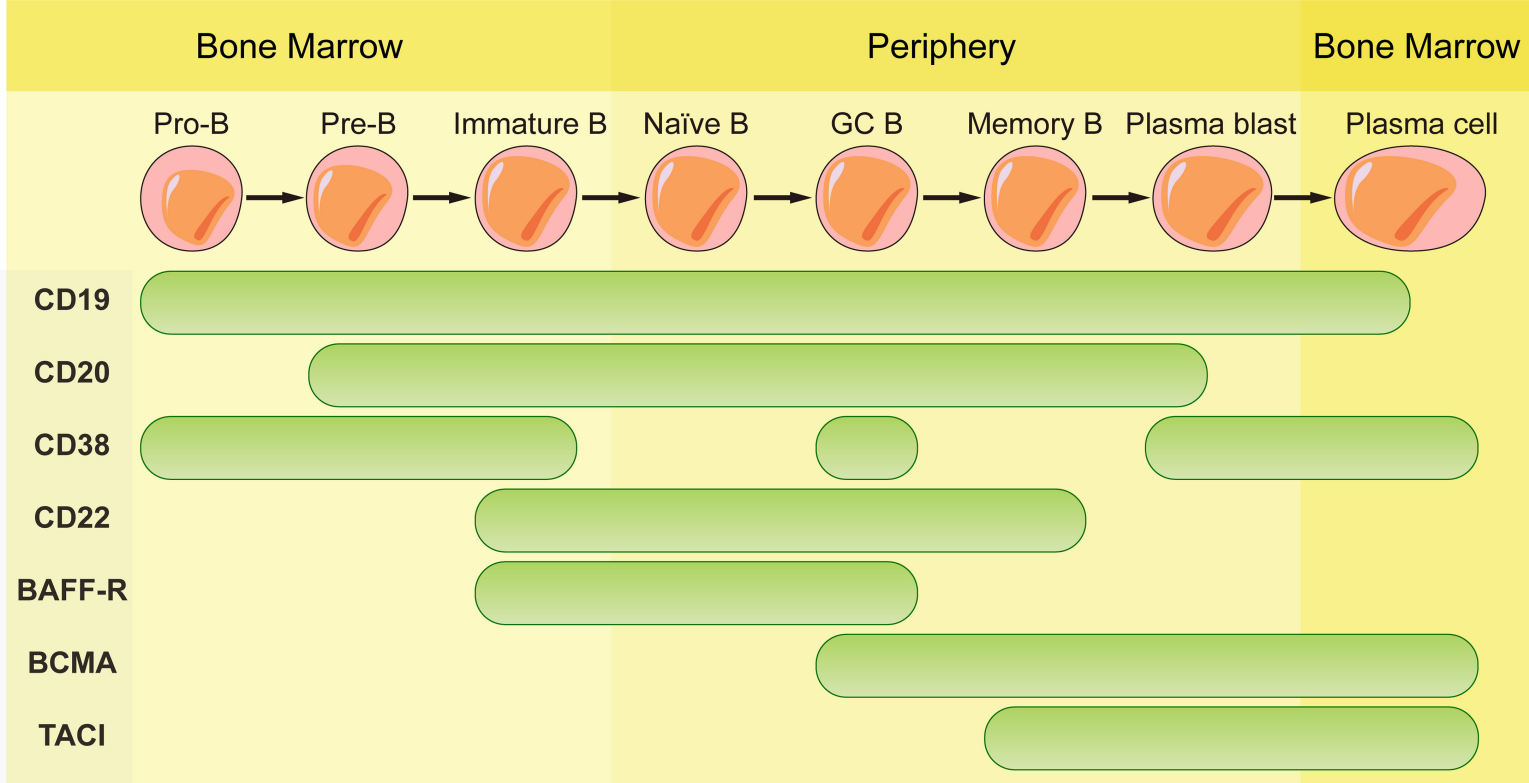
Schematic representation of CD19, CD20, CD38, CD22, BAFF-R, BCMA and TACI expression according to B cell maturation steps.

The mAbs used in field of autoimmune diseases mainly target CD20, CD19, CD22, CD38 and B-cell activating factor (BAFF). They are expressed differently during the development of B cell lineage (**Figure 1**). Some mAbs were approved by FDA for the treatment of autoimmune diseases (**Table 1**).

**Table 1:** FDA approved mAbs in autoimmune diseases.

Target and technology	Drug	FDA-approved diseases
Chimeric anti-CD20 mAb	Rituximab	Rheumatoid arthritis; Granulomatosis with polyangiitis; Microscopic polyangiitis; Pemphigus
Humanized anti-CD20 mAb	Ocrelizumab	Relapsing and progressive multiple sclerosis
Fully human anti-CD20 mAb	Ofatumumab	Relapsing multiple sclerosis
Humanized anti-CD19 mAb	Inebilizumab	Neuromyelitis optica spectrum disorder
Fully human anti-BAFF mAb	Belimumab	SLE; Lupus nephritis

### 3.1 CD20

Antibodies targeting CD20 which is expressed by B cells at almost all stages of development except for pro-B cells, plasmablasts and plasma cells are currently the most widely used mAbs. Although these antibodies have the same target, their structures and indications are very different. The first generation of anti-CD20 mAbs includes murine and chimeric mAbs represented by rituximab. Rituximab is chimeric mAb with 34% mouse protein in the variable region, resulting in lower incidence of human anti-mouse antibody (HAMA) reaction than murine mAbs. They have shown high efficiency in pemphigus, RA, granulomatosis with polyangiitis and microscopic polyangiitis (21–23). The patients with good clinical responses had sustained decrease in anti-dsDNA antibodies and anti-CCP autoantibodies (24–27). High frequency of memory B cells was related to poor clinical response to rituximab (28). However, two randomized controlled trials testing rituximab in lupus nephritis and extra-renal lupus failed to achieve their primary endpoints. There was no significant difference of the proportion of patients achieving complete or partial response between the placebo and the treatment arms. The reason of the failure may be associated with the usage of high doses of glucocorticoids and immunosuppressive therapy, patient heterogeneity, the study size, etc. (29, 30). Meanwhile, rituximab treatment decreases patients’ humoral immune response to recall antigens that will increase the risk of infection and long-term expansion of uncontrolled tumor cells (31, 32).

Recently, 2^nd^ generation of anti-CD20 agents including humanized mAbs and fully human mAbs were developed to reduce immunogenicity and prolong the half-life after infusion into patients. Ocrelizumab, ofatumumab and veltuzumab belonged to this generation were put into use one after another. They were confirmed to bind to the Fc receptor on B cells tighter (33). Ocrelizumab, the humanized mAb, was proved to be efficient and approved by FDA in relapsing MS and primary progressive MS (34, 35) since patients with progressive MS have few treatment options. Compared with rituximab, it induces greater extent of ADCC and lesser extent CDC (36, 37). Relapsing-remitting MS (RRMS) patients treated with ocrelizumab got 46-47% lower annualized relapse rate and 94-95% reduction of active lesions (38). Moreover, ocrelizumab begun to show better efficacy in younger patients with progressive form of MS (39). Ofatumumab is fully human mAb with completely removal of murine components. It was approved by FDA for the treatment of relapsing forms of MS in 2020. In the trials compared to teriflunomide, patients with ofatumumab treatment showed lower annualized relapse rate (40). In addition, it is the first self-administered B cell depletion option in MS which can be delivered *via* autoinjector pen and avoid patients’ visit to infusion center (41).

3^rd^ generation of anti-CD20 mAbs contains glycoengineered Fc portion which increases affinity to Fc receptor III on innate immune effector cells such as NK cells, macrophages, and neutrophils which could remove the antibody-coated cells. This category is represented by obinutuzumab, a humanized anti-CD20 mAb which is more efficient at eliminating organ resident B cells by inducing signaling-dependent B cell death (42). Obinutuzumab binds to different epitope from rituximab and does not induce CD20 clustering or antibody internalization. Therefore, greater efficacy and less resistance are observed (43).

### 3.2 CD22

CD22 is expressed on developing B cells except for plasmablasts and plasma cells (44). The phase III data of epratuzumab which is a humanized mAb targeting CD22 indicated no differences compared with standard therapy in patients with SLE (45, 46). Epratuzumab decreased activation of B cell receptor and depleted only part of B cells. Low CD22 expression and low binding with epratuzumab of CD27^+^ memory B cells resulted in the failure of the therapy.

### 3.3 BAFF

BAFF is a B cell survival factor, resulting in expanded B cell compartment and relaxed negative selection within the GC (47–49). Autoimmune diseases can be induced by overexpression of BAFF in mouse model and elevated serum BAFF levels are found in patients with systemic sclerosis (47, 50). Dysregulated expression of BAFF contributes to autoimmune diseases through its effects on activation, proliferation, survival and immunoglobulin secretion of B cells (51). Belimumab, the only one approved biologic targeting B cells for SLE, is a fully human anti-BAFF mAb. Belimumab prevents BAFF from signaling through receptors (BAFF receptor, TACI, and BCMA) on B cells by binding to it. BAFF receptor (BAFF-R) is expressed on the surface of human peripheral B cell subsets except PCs and centroblasts in the dark zone of GCs (52). BCMA is expressed on long-lived plasma cells while TACI is expressed by plasma cells, activated B cells, marginal zone B cells and switched memory B cells (53). Since BAFF-R is the major receptor for BAFF-dependent response in peripheral blood, belimumab reduces naïve B cells and B cells at early developmental stages rapidly while B cells of later stages such as PCs and memory B cells exhibit resistance due to the lack of BAFF-R (54).

### 3.4 CD19

Antibodies are produced by both short-lived plasma cells and long-lived plasma cells. Unlike short-lived plasma cells, nondividing long-lived plasma cells are always preserved after the treatment of conventional immunosuppressive drugs or mAbs for B cells’ depletion (55).

Since CD19 is expressed on whole B cell development stages as well as one subpopulation of last differentiation stage, plasma cells (56), using CD19 as target to eliminate B cells is theoretically more effective than CD20. Inebilizumab is a humanized anti-CD19 mAb approved by FDA for neuromyelitis optica spectrum disorder (NMOSD), a rare relapsing autoimmune disease of the CNS that causes paralysis and blindness (57). Inebilizumab is still effective in patients previously been treated with rituximab (58). However, treatment with Obexelimab which is a fully human anti-CD19 antibody in SLE patients didn’t reach defined endpoints and the clinical trials was stopped at phase II. Thus, anti-CD19 mAbs didn’t obtain comparable effect in autoimmune diseases as CART cells targeting CD19.

### 3.5 CD38

CD38 is a glycoprotein with ectoenzymatic functions which is expressed on plasmablasts, short-lived and long-lived plasma cells and weakly expressed on other lymphoid cells. The expression of CD38 on long-lived plasma cells makes it a favorable target for depletion of antibody-producing plasma cells. However, its expression on other immune cells such as macrophages, T cells and NK cells may result in side effects in the immune system. The functions of CD38 include cellular adhesion and migration as well as enzymatic activity. As an ADP-ribosyl-cyclase, it can convert cellular NAD to cyclic ADP ribosyl (cADPR) and nicotinamide (NAM) and convert cADPR to ADP-ribose as a hydrolase (59, 60). There is no anti-CD38 mAb approved by FDA in autoimmune diseases at present. Daratumumab, a fully human anti-CD38 mAb approved for treatment in multiple myeloma was used to treat patients with refractory SLE (61, 62). Significant depletion of long-lived plasma cells was observed, and the level of autoantibody reduction was comparable with that observed after the treatment of bortezomib, without toxic effects. Even so, these findings still need to be confirmed in more patients. Interestingly, reduced expression of CD38 was found on the remaining plasma cells after the daratumumab-treatment (63). This transient and general phenomenon is also observed in patients with multiple myeloma which are restored to baseline levels months after the last infusion of daratumumab (64).

However, therapies depleting all long-lived plasma cells are unsafe since they will deplete plasma cells that secrete protective antibodies as well as plasma cells secreting pathogenic antibodies. Qingyu et al. labeled plasma cells with a conjugate of an antibody recognizing plasma cells with the antigen, OVA in murine model. This proof-of-principle study can isolate and deplete OVA-specific plasma cells according to their secreted molecules with drop in the related serum antibody levels (65). It gives a possible solution for depleting specific plasma cells in the future.

## 4 CAR-based therapies in autoimmune diseases

CAR is generated by connecting intracellular signaling endo-domain with extracellular antigen-recognition domain which can be derived from mAb in the form of a single-chain Ab fragment (scFv) including variable heavy (V_H_) and light (V_L_) chains. The antigen-specific recognition domain fused to T cell signaling machinery can be changed according to specific cell-types we want to target and allows T cells to engage the antigen expressed by target cells in an MHC-independent manner. Upon engagement, CART cells and target cells form non-classical immune synapses which are required for their effector functions. After that, the CAR molecules activate the endo-domain signaling and induce the lysis of the engaged target cells through perforin and granzyme axis, the Fas and Fas ligand axis and the release of cytokines (66). As engineered-T cells armed with CARs have shown significant efficacy in the field of hematological malignancies, they have been adopted to eliminate B cells or plasma cells producing autoantibodies in autoimmune diseases. In addition to CART cells, treatments modified based on the theory of CAR such as, chimeric auto-antibody receptor (CAAR) T cells and CAR-Tregs are also introduced into field of autoimmune diseases (**Table 2**).

**Table 2:** Currently employed CAR-based therapies in autoimmune diseases.

Interventions	Locations	Clinical Trials. Gov identifier	Therapeutic indications	Phase
4SCAR T cells (targeting CD19, BCMA, CD138 and BAFF-R)	China, Guangdong; China, Guangxi	NCT05459870	Autoimmune diseases	Phase 2
CD7 CAR T cells	China, Zhejiang	NCT05239702	Crohn diseases; Ulcerative colitis; Dermatomyositis; Still disease; Autoimmune diseases	Early Phase 1
CD19/BCMA CAR T cells	China, Zhejiang	NCT05030779	Systemic lupus erythematosus; Autoimmune diseases	Early Phase 1
CD19/BCMA CAR T cells	China, Zhejiang	NCT05085418	Immune nephritis; Autoimmune diseases; Lupus nephritis	Early Phase 1
CD19/BCMA CAR T cells	China, Zhejiang	NCT05263817	POEMS syndrome; Amyloidosis; Autoimmune hemolytic anemia; Vasculitis	Early Phase 1
CT103A cells (targeting BCMA)	China, Hubei	NCT04561557	Autoimmune diseases; Autoimmune diseases of the nervous system; Neuromyelitis optica spectrum disorder; Myasthenia gravis; Chronic inflammatory demyelinating polyradiculoneuropathy; Immune-mediated necrotizing myopathy	Early Phase 1
CD19 CAR T cells	China, Shanghai	NCT03030976	Systemic lupus erythematosus	Phase 1
tanCART19/20 (targeting CD19 and CD20)	China, Beijing	NCT03605238	Neuromyelitis optica spectrum disorder	Phase 1
BCMA-CD19 cCAR T cells	China, Guangdong	NCT05474885	Relapsed/Refractory systemic lupus erythematosus	Phase 1
Descartes-08 CAR T cells (targeting BCMA)	United States, California; United States, Florida; United States, Georgia; United States, North Carolina; United States, Oregon;	NCT04146051	Generalized myasthenia gravis	Phase 2
DSG3-CAART cells	United States, California; United States, Illinois; United States, lowa; United States, New York; United States, North Carolina; United States, Pennsylvania; United States, Texas; United States, Washington	NCT04422912	Mucosal-dominant pemphigus vulgaris	Phase 1
MuSK-CAART cells	United States, California;	NCT05451212	MuSK myasthenia gravis	Phase 1

### 4.1 CART cells

In NZB/W and MRL-lpr lupus-like mouse models, CART cells targeting CD19 successfully eliminated aberrant CD19^+^ B cells and induced remission with decline in total IgM and IgG antibodies as well as anti-DNA IgG and IgM. Additionally, the mice showed improvement in lupus nephritis and prolonged life span. Anti-CD19 CART cells could actively eliminate CD19^+^ B cells up to 11 months and CD19^+^ B cell aplasia existed during the treatment (67). Moreover, this technique has already shown promising effect and tolerable in patients with autoimmune diseases. Five patients with refractory SLE were enrolled in a compassionate-use CART program. Drug-free remission of diseases was achieved in all patients three months after anti-CD19 CART cells administration and even after the reappearance of B cells. The treatment was well tolerated with only mild cytokine release syndrome (CRS) in patients (68). However, CD19 targeting CART cells can not eliminate long-lived plasma cells completely as not all of them express CD19, resulting in inadequate treatment for antibody-mediated autoimmune disease (69).

The Center for Drug Evaluation (CDE) of China’s National Medical Products Administration (NMPA) has approved its investigational new drug (IND) application for the new extended indication of NMOSD for a fully human BCMA CART cell injection (Equecabtagene Autoleucel, CT103A). Twelve relapsed/refractory NMOSD patients with AQP4-IgG who had at least one year of treatment with at least one immunosuppressant were included in the investigator-initiated clinical study. The data showed that the Equecabtagene Autoleucel injection was safe as no immune effector cell-associated neurotoxicity syndrome (ICANS) events. It can reduce the disability score and improve the functions of sensory, nervous, and motor systems, providing a proof-of-concept for CART cells therapy to treat NMOSD caused by AQP4 produced by plasma cells (70).

### 4.2 CAAR T cells

To identify cells secreting antibodies such as autoreactive B cells, the researchers generated CAAR T cells by replacing the extracellular antigen-recognition domain with a specific antigen which could recognize and bind to the target autoantibodies expressed on autoreactive cells. This modification of CART cells eliminates surface immunoglobulin memory B cells directly and short-lived plasma cells that produce autoantibodies indirectly. The pathogenic B cells in a mouse model of pemphigus vulgaris (PV) produce antibodies against desmoglein (Dsg) 3. Ellebrecht et al. engineered T cells to express Dsg3 which can be recognized by pathogenic B cells with anti-Dsg3 B cell receptors on surface and then, destroy pathogenic B cells specifically even in the presence of soluble serum anti-Dsg3 IgG (71). Limited PV growth and decreased Dsg3 serum antibody levels were observed in patients without any toxic off-target activity. Conclusively, this study indicated that CAAR T cells can be applied in antibody-mediated autoimmune diseases as a promising therapeutic option.

### 4.3 CAR Treg cells

Since the autoimmune diseases are caused by the loss of immune tolerance, Tregs with immunosuppressive characteristics were transformed to CAR-Tregs to provide a promising option to restore the immune system (72, 73) and fight against autoimmune diseases. In addition to production of granzymes and perforin to destroy target cells, they secret inhibitory cytokines, such as IL-10 and TGFβ. They can also consume IL-2 using CD25 receptor and prevent the activation of effector T cells (74–76). However, the low rate of Tregs in peripheral blood limits their application. To increase the number of Tregs for CAR transduction, Tenspolde et al. introduced Foxp3 gene into CD4^+^ effector T cells which prevented Tregs from being transformed or differentiated into other cells (77). Nevertheless, the insulin-specific CAR-Tregs didn’t prevent NOD/Ltj female mice from diabetes even though these CAR-Tregs can still be detected four months after the infusion. The ineffectiveness may be attributed to the diversity of insulin structure. Another study converted CD4^+^ T cells into myelin oligodendrocyte glycoprotein (MOG) CAR-Tregs by transducing MOG CAR gene and Foxp3 gene in the mouse model to treat MS. The connection of CAR-MOG receptor brought Tregs to MOG^+^ oligodendrocytes closely to prevent immune attacks against them. Ten days upon the infusion, the results revealed that the MOG CAR-Tregs could suppress the proliferation of effector T cells, decrease IL-12 and IFN levels and protect mice against EAE inflammation (78). However, it should be noted that the instability of Tregs might convert their immunosuppressive manner into effector function when they enter different inflammation zones.

## 5 Discussion

### 5.1 The advantages and disadvantages of both approaches

**Figure 2:**
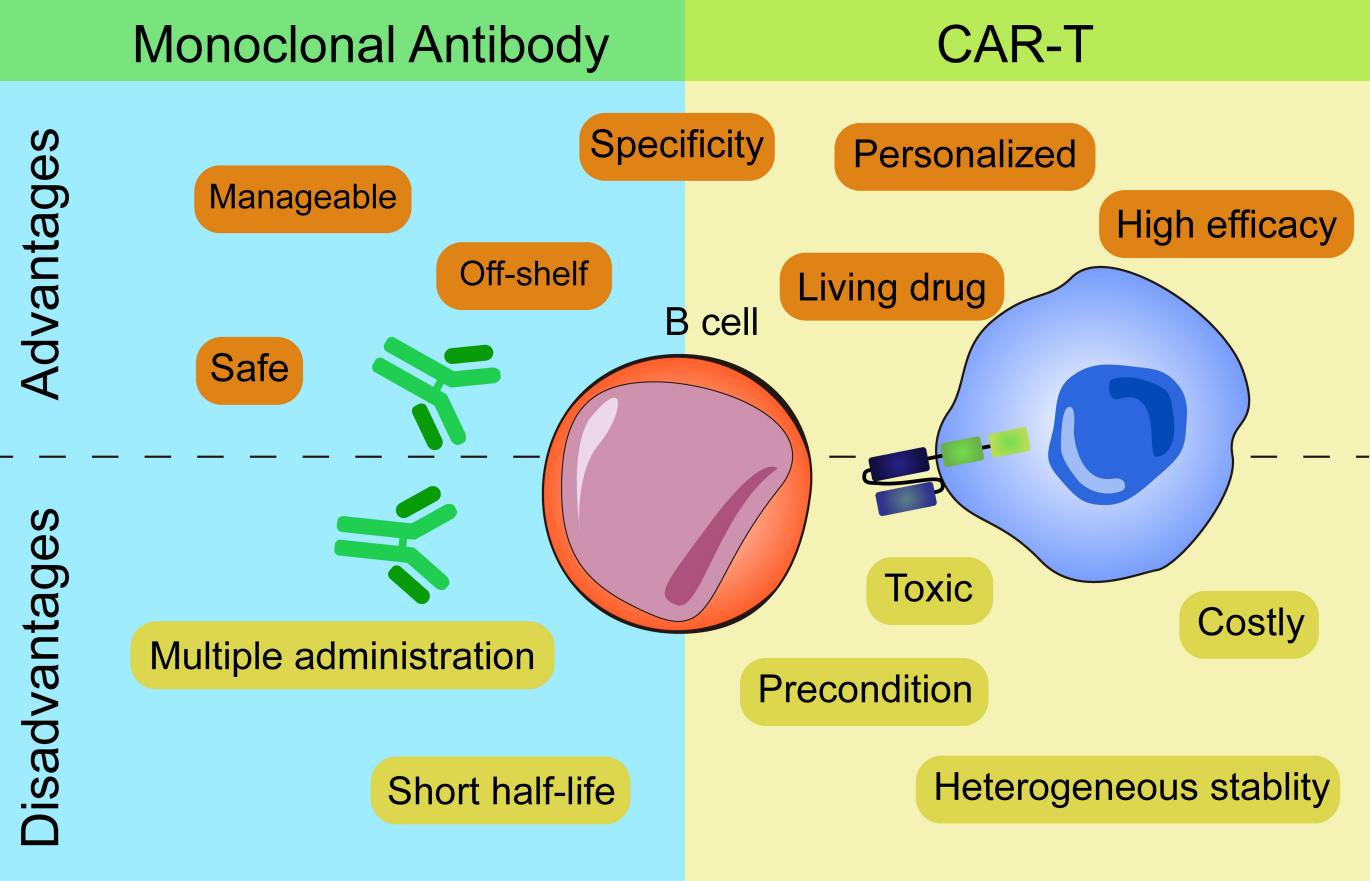
Advantages and disadvantages of mAbs and CAR-based therapies.

The above two types of immunotherapies both have advantages and disadvantages from the process of production to the clinical application (**Figure 2**).

In terms of production, CART cells which are personalized medicines need to be manufactured for each single patient while mAbs are off-shelf reagents. Individual production of CART cells leads to higher costs and the stability and persistence of T cells vary from person to person. CART cells of several patients perform insufficient ex vivo proliferation, expansion and persistence that result in unstable clinical efficacy.

As for administration frequency, mAbs need multiple administrations to achieve the desired effect due to their short half-life. In contrast, CART cells, as a “living drug”, can proliferate and expand *in vivo* after infusion and persist for a long time. But CART cells need lymphodepletion with fludarabine and cyclophosphamide before the administration while mAbs don’t need. Moreover, the ‘armored CART’ concept enables the cells to express various proteins (cytokines, antibody-like protein…) that makes it easy to combine therapies. An example of this engineering is that Marcela et al. combined CAR and antibodies in one cell by introducing CART- Blinatumomab (BiTE) cells which efficiently killed both EGFRvIII^+^ and EGFRvIII^-^ glioma cells (79). BiTE, the bispecific antibodies which can redirect T cells to CD19^+^ target cells have been proved efficient in ALL patients (80). Secretion of BiTE continuously and activation of bystander T cells make CART.BiTE cells a dual-targeted platform to prevent antigen escape. Take inspiration from this, CART.BiTE targeting B cells’ antigen provides a new solution for autoimmune diseases in the future.

When applied in clinic, depletion of B cells by mAbs has limited therapeutic efficacy as they can’t access autoreactive B cells within lymphatic organs and inflamed tissues (81, 82). Then, mAbs can hardly deplete B cell completely. Even CART cells are better in this regard, immunosuppressive cells or molecules *in vivo* including Bregs secreting IL-10 or PD-1 etc. might affect the killing efficiency of CART cells as well (83). When antigen loss appear, CART cells are superior to mAbs as target cells with low antigen expression will escape recognition by mAbs. mAbs need high numbers of antigen molecule to efficiently activate either ADCC or CDC (84, 85). However, this merit of CART cells has its caveats that greater efficacy usually comes with toxicities which can’t be necessarily anticipated from previous and safe use of mAbs specific for the same target, due to the intrinsic functional activity of T cells to which the CAR molecules are engaged. To balance the safety and effectiveness of CART cells at the same time, dosing strategy is another concern. If this balance is not well controlled, CART cells will lead to lethal toxicities that result from CRS and neurotoxicity in patients.

In recent years, B cell depletion therapies occupy an increasingly important position in the treatment for autoimmune diseases. The clinical applications of the mAbs targeting B cells and plasma cells are effective in a broad range of diseases, emphasizing the importance of B lineage cells in the pathogenesis of autoimmune diseases. The adoption of CAR-based therapies from hematological malignancies brings the treatment of autoimmune diseases into a new era. However, larger cohorts are needed for evaluation of CAR-based therapies before broadly application in patients, even some clinical trials and cases have already proven its effectiveness in a subset of diseases. Current treatments all have their own advantages and disadvantages. New therapeutic approaches are emerging as we understand the mechanisms deeper and exploit more targets, giving clinicians more options tailored to each patient’s wishes and status.

## Author contributions

ZZ wrote and edited the review. QX edited the review and arranged the figures and table. LH conceived and edited the review. All authors contributed to the article and approved the submitted version.
